# Surface Tension and Viscosity Dependence of Slip Length over Irregularly Structured Superhydrophobic Surfaces

**DOI:** 10.1021/acs.langmuir.2c01323

**Published:** 2022-09-20

**Authors:** Linsheng Zhang, Yasmin A. Mehanna, Colin R. Crick, Robert J. Poole

**Affiliations:** † School of Engineering, University of Liverpool, Liverpool L69 3GH, United Kingdom; ‡ Materials Innovation Factory, Department of Chemistry, University of Liverpool, Liverpool L69 7ZD, United Kingdom; § School of Engineering and Materials Science, Queen Mary University of London, London E1 4NS, United Kingdom

## Abstract

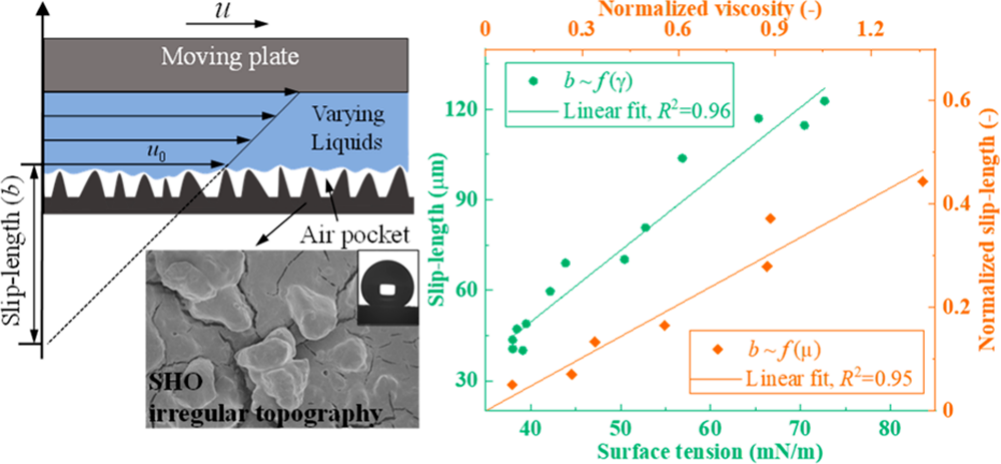

A comprehensive understanding of the slip phenomenon on liquid/solid interfaces is essential for multiple real-world applications of superhydrophobic materials, especially those involving drag reduction. In the current contribution, the so-called “slip-length” on an irregularly structured superhydrophobic surface was systematically evaluated, with respect to varying liquid surface tension and viscosity. The superhydrophobic polymer–nanoparticle composite (SPNC) material used exhibits a dual-scale surface roughness and was fabricated via coating a surface with a mixture of polydimethylsiloxane solution and functionalized silica particles. A cone-and-plate rheometric device was employed to quantify the slip length. To independently study the impact of surface tension and viscosity, three types of aqueous solutions were used: sodium dodecyl sulfate, ethanol, and polyethylene glycol. Our experimental results demonstrate that a decreasing surface tension results in a decreasing slip length when the fluid viscosity is held constant. Meanwhile, the slip length is shown to increase with increasing viscosity when the surface tension of the various liquids is matched to isolate effects. The study reveals a linear relationship between slip length and both capillary length and viscosity providing a reference to potentially predict the degree of achievable drag reduction for differing fluids on SPNC surfaces.

## 1 Introduction

The concept of slip length was first proposed two centuries ago,^1^ at which time the no-slip boundary condition was universally accepted for a solid/liquid interface in fluid dynamics. During the last two decades, nanoscale and microscale fluidic technologies have been developed,^2^ such that the inherent assumption of a no-slip boundary condition was revisited throughout the literature,^3,4^ especially in the scenario where solid surfaces have been coated or treated to become so-called “superhydrophobic” (hereafter referred to as “SHO” surfaces).^5^ The large slip obtained from liquid/solid interfaces enables the SHO surfaces to potentially reduce drag in a flow, and the slip length, in return, is an ideal parameter to quantify the degree of drag reduction.^6^

As an area-average quantity,^7^ the apparent slip originates from the various “air pockets” (often termed “plastron”) trapped on the nanogeometrical and microgeometrical roughness features of a SHO surface (i.e., nonwetted state) since the shear stress is much lower at an air/liquid interface than at a solid/liquid interface.^8,9^ Therefore, the air pockets that are generally associated with the structural pattern of SHO surfaces are of fundamental importance for a slip boundary condition to occur.^10^ Numerous studies^11−15^ have focused on the topography of SHO surfaces, which aim to predict the slip length with a given structure^14^ or optimize the surface features for a higher slip length.^15^ However, the test liquids for most of these studies are restricted to either water^16,17^ or water–glycerol (to enhance the viscosity)^18,19^ and the systematic impacts of *isolated* liquid property variation on slip length have been largely overlooked.^20^ Recently, unexpectedly low slip lengths were found to be the result of a nonuniform surface tension over the formed air pockets on SHO surfaces made from hydrophobic polydimethylsiloxane (PDMS) and comprised of regular rectangular gratings,^21^ which can be caused by the accumulation of matter (such as microparticles, surfactants, or other “contaminants” such as velocimetry tracking particles^22^). Previous theoretical works^23,24^ also hypothesized that the adsorption of surfactants on the air/liquid interface would immobilize the interface and, hence, modify the slip length. Moreover, any local slip has been observed to vanish because of the decreasing surface tension of the given fluid and the induced Marangoni forces along the microfeatures.^25^ Thus, there is evidence that surface tension modification may eliminate any potential slip/drag reduction for some SHO surfaces comprised of certain topologies. In addition to surface tension, shear viscosity also has a direct influence on slip length. On a given SHO surface, the slip length was shown to increase at the same ratio as shear viscosity increment by using water and 30 wt % glycerine.^18,19^ Once again, there is little systematic work investigating isolated effects of viscosity (while holding surface tension constant) for other fluids and SHO surfaces.

**Figure 1:**
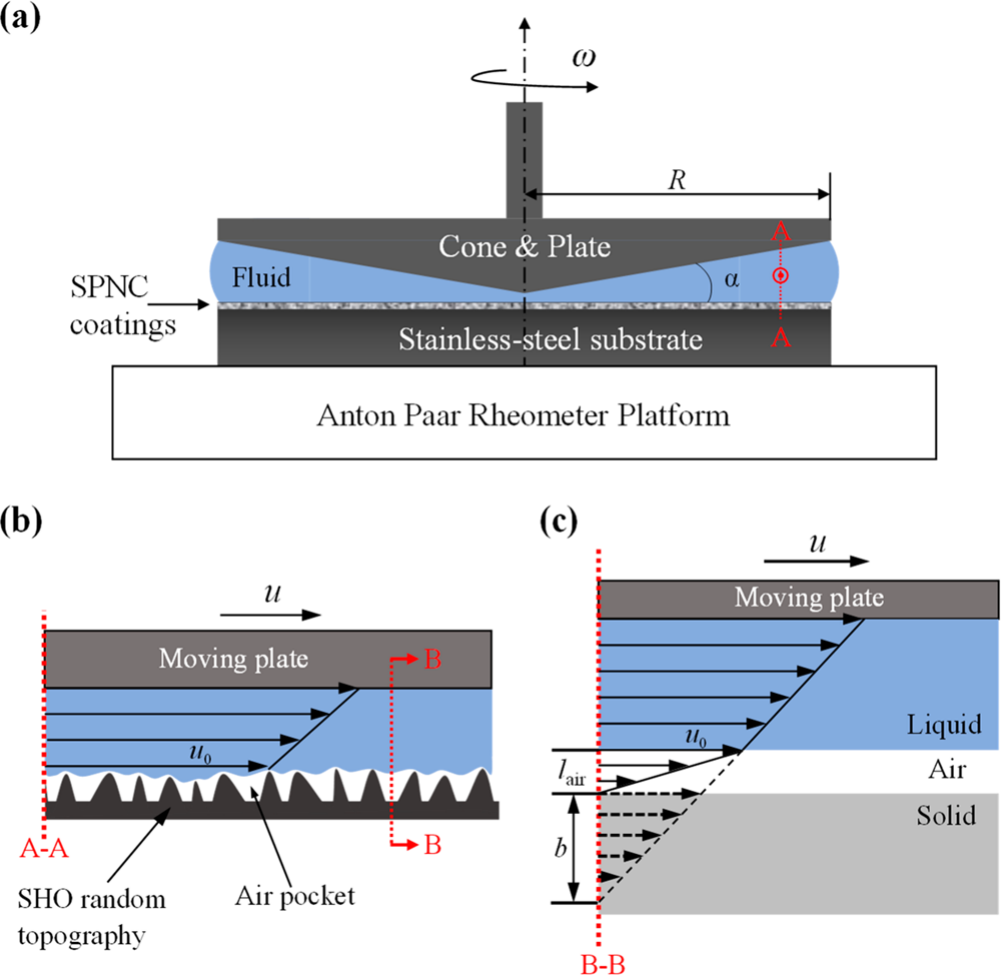
Slip-length measurement setup and concept of apparent slip on SHO surfaces. (a) Schematic diagram of slip-length measurement on the SPNC surface with a cone-and-plate rheometer system, where ω is the angular velocity and α is the angle of the cone. The SPNC surface was coated on a stainless-steel substrate with the same radius (*R*) as the cone. (b) An illustration of cross-sectional view at line A-A in panel (a), where *u* is the velocity of the moving plate and *u*_0_ is the slip velocity. Air pockets of different volumes are formed on the SPNC surfaces as shown in the schematic. (c) A conceptual illustration of the slip-length concept based on an idealized air layer (can also be seen as the cross-sectional view of B–B in panel (b)), where *l*_air_ is the thickness of the air layer and *b* is the slip length.

In this work, the surface tension and shear viscosity dependences of slip length on an irregularly structured SHO surface (so-called “SPNC” surfaces) were experimentally studied. A SPNC surface is a recently developed superhydrophobic polymer–nanoparticle composite coating, which features extreme superhydrophobicity and excellent resilience.^26,27^ A Couette flow based on a rheometric cone-and-plate system was employed to measure the slip length (Figure 1a). This technique and geometry has been previously used by Choi and Kim^18^ and Xu et al.,^28^ and similar experiments for slip-length measurement have also been conducted based on a parallel-plate geometry^29^ and Taylor–Couette flow.^30^ A schematic of the irregular microstructures of a SPNC surface and the Couette flow over it can be found in Figure 1b. The volumes of individual air pockets are assumed to vary dependent on the size of the local microfeatures. Cross-sectionally viewing the B–B location in Figure 1b, an idealized flow above a pure air layer can be postulated (Figure 1c). The shear stress (τ_*i*_) between such an idealized air–liquid interface can be expressed as
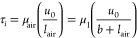
1where *u*_0_ is the slip velocity, *b* is the slip length, *l*_air_ is the thickness of the air layer (which is assumed to be flowing uniformly), and μ_air_ and μ_1_ are the viscosities of air and liquid, respectively.

Therefore, the slip length (*b*) can be obtained theoretically via eq 2:^31^
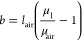
2

An alternative model uses an assumption that there is a net-zero mass flow rate within the surface layer flow,^24,32,33^ such that there is a recirculating flow in the air layer. In this situation, eq 2 is found to be modified by a factor of ^1^/_4_ in the viscosity ratio:
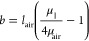
3Equations 2 and 3 show that the slip length is determined by the air layer thickness (*l*_air_) and shear viscosity ratio (μ_l_ /μ_air_) in both idealized viewpoints (note that it has been recognized that eqs 2 and 3 may, in fact, be more complex for heterogeneous surfaces, such as that studied here, and theoretical studies have attempted to relate viscosity contrast to the topography of the underlying texture^34^). To investigate the validity of these equations, in this study, several liquids (sodium dodecyl sulfate, ethanol, and polyethylene glycol solutions) are utilized to monitor the slip length on SPNC surfaces. The surface tension and shear viscosity dependence of slip length were individually analyzed. The results should help to broaden the application of SHO surfaces to liquids other than water and aid in a better understanding of the effects of varying liquid properties on achievable slip lengths (and, consequently, drag reduction).

## 2 Materials and Method

### 2.1 Manufacture of SPNC Surfaces

As reported in detail in a previous study,^27^ SPNC coatings involve a precoating process (spraying with an adhesive layer) to promote higher robustness, which is achieved by spreading 12 drops (∼220 μL) of adhesion promoter (CYN20, Everbuild Building Products, Ltd.) manually over the substrates (which are made of 304 stainless steel in the current study) and then spraying ∼8 mL of PDMS (Ellsworth Adhesive, Ltd.) solution onto the substrate. The precoated substrates were allowed to partially cure by heating to 50 °C for 15 min. Afterward, the substrates were moved to a 120 °C-adjusted hot plate to spray ∼13 mL of the PDMS/silica mixture for another layer of coating. The coated substrates were left to fully dry on the 120 °C hot plate for 30 min.

PDMS solutions for precoating were prepared by mixing two parts of a Sylgard-186 silicone elastomer (PDMS and a silicon-based curing agent combined with a ratio of 10:1, total polymer mass = 0.5 g), adding hexane (70 mL), and magnetic stirring until dissolved. Hydrophobized silica nanoparticles (0.25 g) were added to 50 mL of the PDMS solution and stirred at room temperature for an hour to obtain the PDMS/silica mixture.

### 2.2 Preparation of Test Liquids

Sodium dodecyl sulfate (SDS) (VWR International, Ltd., ≥98% purity) solutions with concentrations from 1 to 10 mM (at 1 mM intervals) were prepared to achieve a full range of surface tensions (from a high surface tension to the lowest surface tension that corresponds to the critical micelle concentration (CMC)), as well as two very-diluted solutions with concentrations of 0.01 mM and 0.1 mM, which can represent “trace” level of surfactants. 0.5 and 0.75 mM SDS solutions were also prepared to accurately match the surface tension with the liquids described below. To alter viscosity, polyethylene glycol (PEG) (Sigma–Aldrich) polymer with a molar mass of 8000 g/mol was employed to obtain varying PEG solutions (1, 2, and 3 wt %). Meanwhile, ethanol solutions (2, 7.5, 16.5, and 25 wt %) were prepared by diluting absolute ethanol (EtOH, Sigma–Aldrich, ≥99.9% purity) to match the surface tension of specific SDS solutions. Distilled water was used as the solvent/dilutant for all the solutions and the preparations were conducted at room temperature.

### 2.3 Scanning Electron Microscopy

The surface topography of SPNC coatings was imaged using a scanning electron microscopy (SEM) system (Hitachi, Model S4800) operating at a 3 kV acceleration voltage and a 3-mA beam current. Images were taken 7.5 or 8.5 mm away from the samples. To improve the electrical conductivity and ensure a good image quality, samples were vacuum sputter-coated by a thin layer of chromium metal prior to the SEM imaging.

### 2.4 Contact Angle and Surface Tension

Contact angles between SPNC surfaces and various test liquids were obtained with a dynamic shape analyzer (Kruss, Model DSA 100E) through the sessile drop method (drop volumes in the range of 15–20 μL). The dynamic contact angle of the present surfaces with distilled water was also measured in the same facility. By injecting and withdrawing distilled water with a rate of 0.1 μL/s, an initial droplet of 5 μL was increased to 10 μL and then decreased back to 5 μL to determine the advancing and receding contact angles. The contact angle hysteresis may then be determined. A Kruss Model K100 force tensiometer was employed to measure the surface tension of liquid samples with a Peltier temperature control unit to keep the temperature of the liquid constant at 20 °C.

### 2.5 Shear Viscosity

A torque-controlled rheometer (Anton Paar, Model MCR 302) was used to determine the viscosities of the prepared liquids for shear rates from 50 to 100 1/s at 20 °C. 2–3 mL of liquid was deposited on the rheometer testing platform via pipettes, and a cone (of radius *R* = 30 mm, angle α = 2°) geometry was adopted to conduct the measurements. The viscosity results were taken as a reference viscosity for the slip-length calculation described in Section 2.6.

### 2.6 Slip Length

Following previous studies,^18,28^ slip lengths on the present surfaces were quantified by the measured torque difference on the inherent no-slip stainless-steel platform of the rheometer and the SPNC surfaces. The torque (*T*) in a reference no-slip system with a cone-and-plate setup and a specific liquid (of viscosity μ) is calculated via
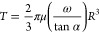
4

The slip length *b* of a SHO surface can be deduced using eq 5 from the torque obtained in the presence of slippage (*T*_s_) when the same liquid is used,
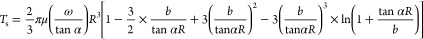
5

Meanwhile, the corresponding drag reduction (DR) on SHO surfaces is defined as
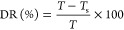
6

An acrylic cone geometry was selected to conduct the slip-length measurement. This acrylic geometry allowed the filled water to be visible so that any wanted bubbles (on the untreated cone) can be detected directly during the measurement. In addition, the curvature of the free surface is easier to check and maintain constant using a transparent cone. The slip lengths with distilled water and SDS solutions were recorded with a shear rate range of 50–100 1/s (Reynolds numbers range of 40–56, which is significantly below the critical Reynolds number for transition to turbulence^35^). These low shear rates also minimize secondary flow effects.^36^ Furthermore, an even lower shear rate (from 30 to 75 1/s with increasing viscosity, to keep the maximum shear stress no more than 0.1 Pa, see the Supporting Information for complete details) was used to avoid any potential surface degradation when those higher viscosity liquids (PEG and EtOH solutions) were applied.^28^ The Reynolds numbers for PEG and EtOH experiments were in the range of 20–47. The shear rate settings and Reynolds numbers for the slip-length measurement with various liquids are shown in Table S1 in the Supporting Information. As shown in Figure 1a, SPNC materials were coated on a circular substrate, which has the same diameter as the cone to minimize any potential edge or free surface effects. The slip lengths were tested at a temperature of 20 ± 0.5 °C as the reference viscosities were obtained in this temperature range.

## 3 Results and Discussion

### 3.1 Surface Morphology and Superhydrophobicity

**Figure 2:**
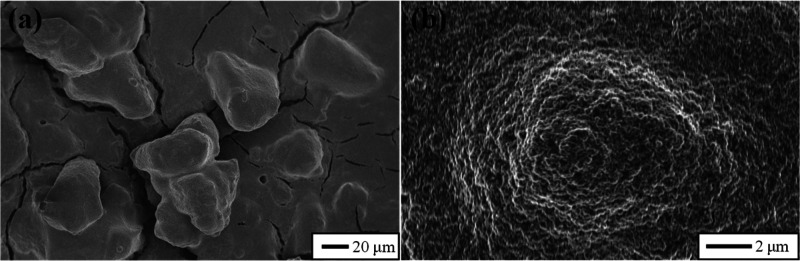
SEM images of SPNC surfaces with different magnifications. Scale bars are shown for each image.

The SEM images, shown in Figure 2, demonstrate a dual-scale surface roughness of the SPNC surfaces. The partial cure of PDMS polymer during the precoating process generated the first-scale surface roughness. Protrusions of 20–30 μm in size and some cracks with different lengths and widths were observed at a lower magnification (Figure 2a). Although the appearance/severity of cracks was observed to be dependent on precise coating operation, experimental conditions were kept as constant as possible in order to reduce variation in the microcrack formation. The second-scale roughness is on the order of several hundreds of nanometers and can be observed in Figure 2b, which results from the arrangement of silica particles. Multiple SEM tests show that microscale feature size remains consistent among the SPNC samples (additional SEM images are available in Figure S1 in the Supporting Information). Further details regarding these SPNC materials have been previously reported.^26,27^

The superhydrophobicity of the current surfaces was confirmed by the large contact angle (158° ± 0.9°) and small contact angle hysteresis (5° ± 0.8°) results with distilled water. Meanwhile, slip length as a novel indicator has also proved an excellent superhydrophobic surface property of SPNC coatings. The slip length ranged from 100 to 160 μm (equivalent to laminar drag reduction ranging from 14%–18%) for 15 different coated samples and ∼30 repeat measurements. An example of the slip-length characterization is shown in Figure S2 in the Supporting Information. Such very high slip-length results are rarely observed in the literature except for a few studies, which were dedicated to its maximization.^12,37^ Following the protocol of previous tests on the resilience of SHO surfaces,^28^ the longevity of this drag reduction over one such SPNC surface was characterized by a long-term slip-length measurement. Results shown in Figure S3 in the Supporting Information demonstrate that the drag-reduction performance of SPNC surfaces remains constant over a period of 10 h (with water flow at a shear rate of 75 1/s).

### 3.2 Liquid Properties

**Figure 3:**
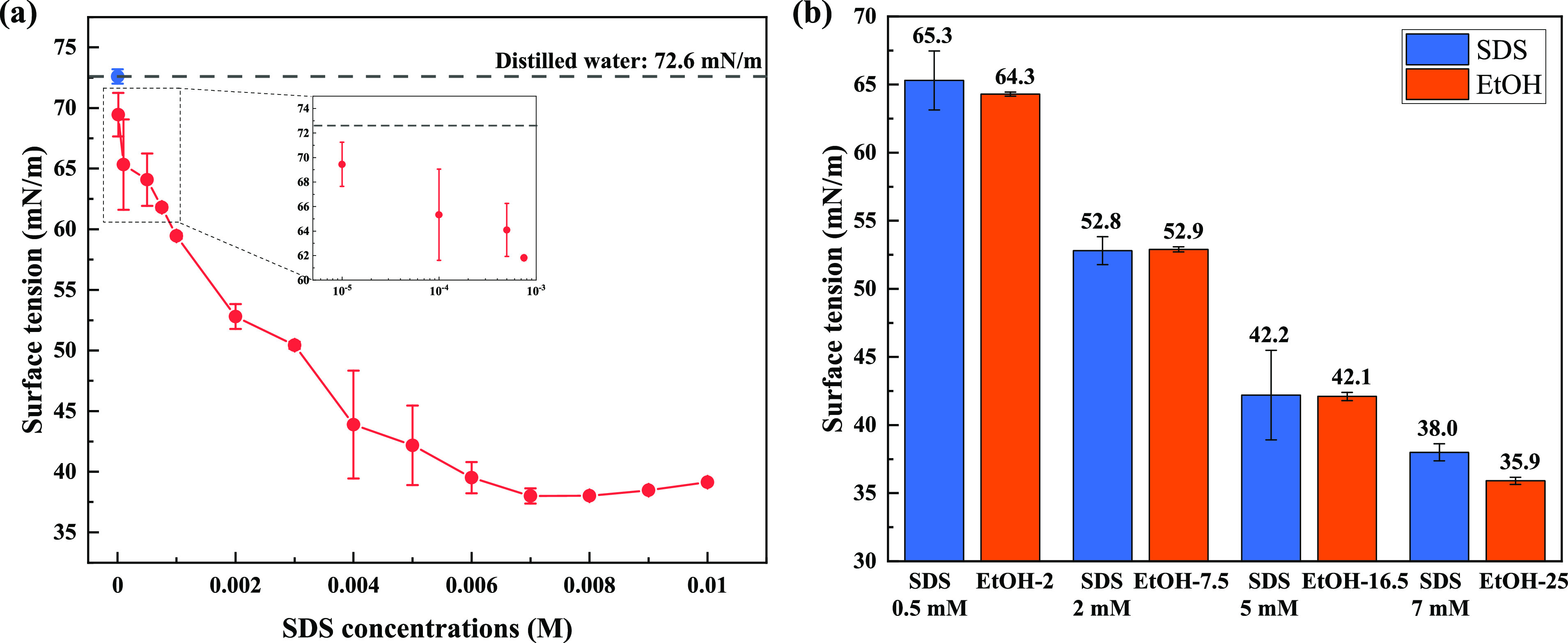
Surface tension results for (a) various SDS solutions and (b) four EtOH solutions selected to match the surface tension of specific SDS solutions. The surface tension of distilled water is shown as a blue filled circle and a dashed line in panel (a), and an expanded view provided in panel (a) shows the surface tension of the “trace” level of SDS solutions. Error bars represent 3δ (3 times the standard deviation) of typically three repeat measurements.

The surface tension of SDS solutions are presented in Figure 3a, as well as the data for distilled water alone (i.e., 0 wt % SDS concentration). The distilled water value (72.6 ± 0.59 mN/m) is consistent with other studies,^38^ confirming the absence of trace contaminants. The surface tension of SDS solutions decreases as the concentration increases until it reaches the CMC (where a minimum surface tension is obtained). CMC is found to be 7 mM in this study which is slightly lower than the results from Hernainz and Caro^39^ where CMC was reported as 8 mM. Note that a very small quantity of surfactant addition also results in a measurable surface tension decrease. As shown in the expanded view in Figure 3a, the “trace” level SDS solutions (0.01 and 0.1 mM) still have measurable surface tension variations, compared to distilled water. Finally, the surface tension results of 0.5 and 0.75 mM SDS solutions, which were prepared to match the surface tension of EtOH and PEG solutions respectively, are also presented in the expanded view in Figure 3a. Four EtOH solutions were selected to match the surface tensions of the various SDS solutions (Figure 3b), together with three PEG solutions (with relatively constant surface tension, as shown in Table 1), were adopted to study the impact of viscosity on slip length (discussed in Section 3.5)

**Table 1:** Surface Tension and Shear Viscosity for PEG and Ethanol Solutions along with the Estimated Uncertainties

sample	concentration (wt.%)	surface tension (mN/m)	shear viscosity (mPa s)
Distilled water	-	72.6 ± 0.59	0.97 ± 0.02
PEG-1	1	63.1 ± 0.08	1.23 ± 0.02
PEG-2	2	62.7 ± 0.11	1.51 ± 0.02
PEG-3	3	61.0 ± 0.11	1.82 ± 0.02
EtOH-2	2	64.3 ± 0.15	1.05 ± 0.02
EtOH-7.5	7.5	52.9 ± 0.19	1.30 ± 0.04
EtOH-16.5	16.5	42.1 ± 0.30	1.83 ± 0.04
EtOH-25	25	35.9 ± 0.27	2.29 ± 0.04

The average viscosity of distilled water was measured to be 0.97 ± 0.02 mPa s at 20 °C, which represents a 3% difference, compared with the result of Kestin et al.^40^ The viscosity of the SDS solutions is taken to be the same as distilled water (the aim of these fluids is to study the impacts of surface tension changes alone on slip length, as discussed in Section 3.4), since there is no measured difference between distilled water and the SDS concentrations up to the CMC. (The viscosity of distilled water and three representative SDS solutions are shown in Figure S4 in the Supporting Information to confirm this). For PEG and EtOH solutions, the viscosity results along with uncertainties are displayed in Table 1 (some data are also shown in Figure 3 for the sake of clearer interpretation).

### 3.3 Changing the Wettability of SHO Surfaces

The wetting behaviors on a SHO surface are of crucial importance regarding achieving apparent slip since a nonwetting condition is a prerequisite for the air-pocket/plastron formation on the topography (i.e., a “Cassie” state). Contact angle (θ) is generally used to characterize the wettability of a surface and is commonly described by Young’s equation,^41^
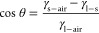
7where γ_s-air_, γ_l-s_, and γ_1-air_ represent the interfacial tension of solid–air, liquid–solid, and liquid–air, respectively.

**Figure 4:**
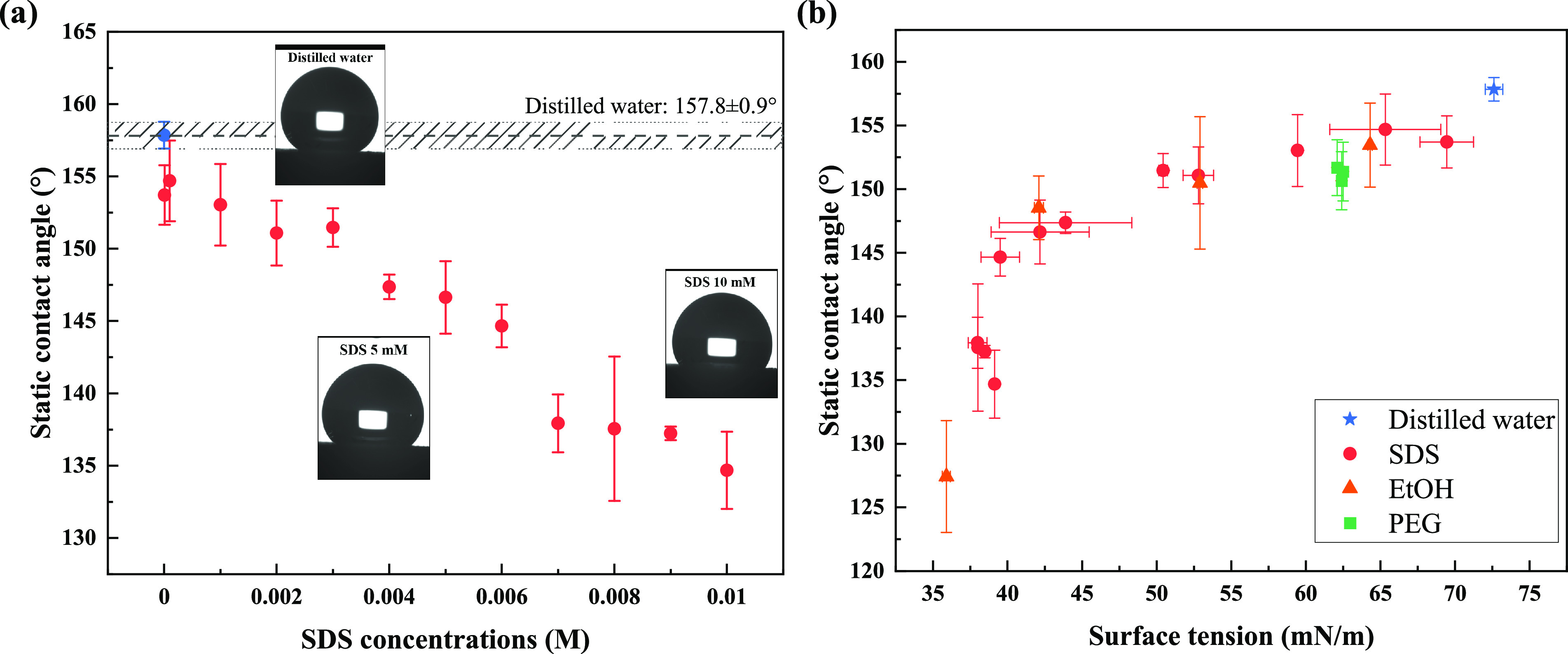
Contact angle measurement on SPNC surfaces. (a) Contact angle values against SDS concentration; the value for distilled water is shown as a blue filled circle and a dashed line (gray region represents the variation of repeats). Representative photographs for contact angle tests with droplets of distilled water, SDS 5 mM and SDS 10 mM solutions are shown. (b) Contact angle values against the surface tensions of various liquid samples in which the contact angle results with EtOH and PEG solutions are plotted along with SDS solutions. The contact angle test was conducted on the same SPNC surface with three fixed locations to apply the droplet and average values are taken. Error bars show 3δ of results from different locations.

Figure 4a shows that the contact angle between the SPNC surface and SDS solutions continuously decreases as the SDS concentration increases and then becomes constant when the SDS concentration reaches the CMC. This is a consistent trend with Figure 3a, which indicates the surface tension determines the contact angle for SDS (as can be interpreted via eq 6). According to a previous study,^42^ SDS as a typical ionic surfactant that contains a linear hydrophobic tail, is defined as a surface tension (*γ*_1-air_) -controlled surfactant, rather than a liquid–solid interfacial tension-controlled (γ_1–s_) surfactant, for which contact angle continuously decay with increasing concentration and SHO surfaces transform to be hydrophilic due to particle adsorption at higher concentrations. The wettability of SPNC surfaces has a tendency to be altered by the SDS solutions as the increasing concentration (i.e., decreasing surface tension). However, SDS does not change the SPNC surfaces to become hydrophilic as the contact angles remain high at a minimum value of 135° above the CMC.

The contact angles are plotted against the surface tension of various liquids in Figure 4b, in which EtOH and PEG solutions are both included. Figure 4b reveals that the surface tension decrease causes an increase in wettability (i.e., decrease in contact angle) for a given surface. This has been shown previously in the literature not only for surfaces focusing on superhydrophocity^42^ but also surfaces with contact angles of <90°.^43^ It is also found that contact angle on SPNC surfaces is shown to be more sensitive to surface tension in the hydrophobic (90° < θ < 150°) region than in the superhydrophobic region (150° < θ < 180°). Furthermore, the surface tension determination on contact angle is consistent with varying aqueous solutions (EtOH and PEG). As discussed in Section 3.2, the surface tensions of the PEG solutions are generally constant (±1.2 mN/m). Therefore, contact angles are also constant with different PEG concentrations, which indicates that the wettability does not change. However, contact angles between SPNC surfaces and EtOH solutions decrease significantly with increasing EtOH concentrations (Figure 4b). This is due to the fact that the addition of ethanol to water decreases the surface tension and, thus, gives rise to an increase wettability of the current surfaces. Other studies^44−46^ have previously reported that the wettability of SHO surfaces are increased and the surfaces more easily wet by ethanol–water solutions than by water alone. SPNC surfaces behave as either superhydrophobic or hydrophobic for all the prepared liquids. Therefore, we would expect the surfaces to reduce drag in aqueous liquid flow and exhibit a nonzero slip length.

### 3.4 Surface Tension Dependence of Slip Length

**Figure 5:**
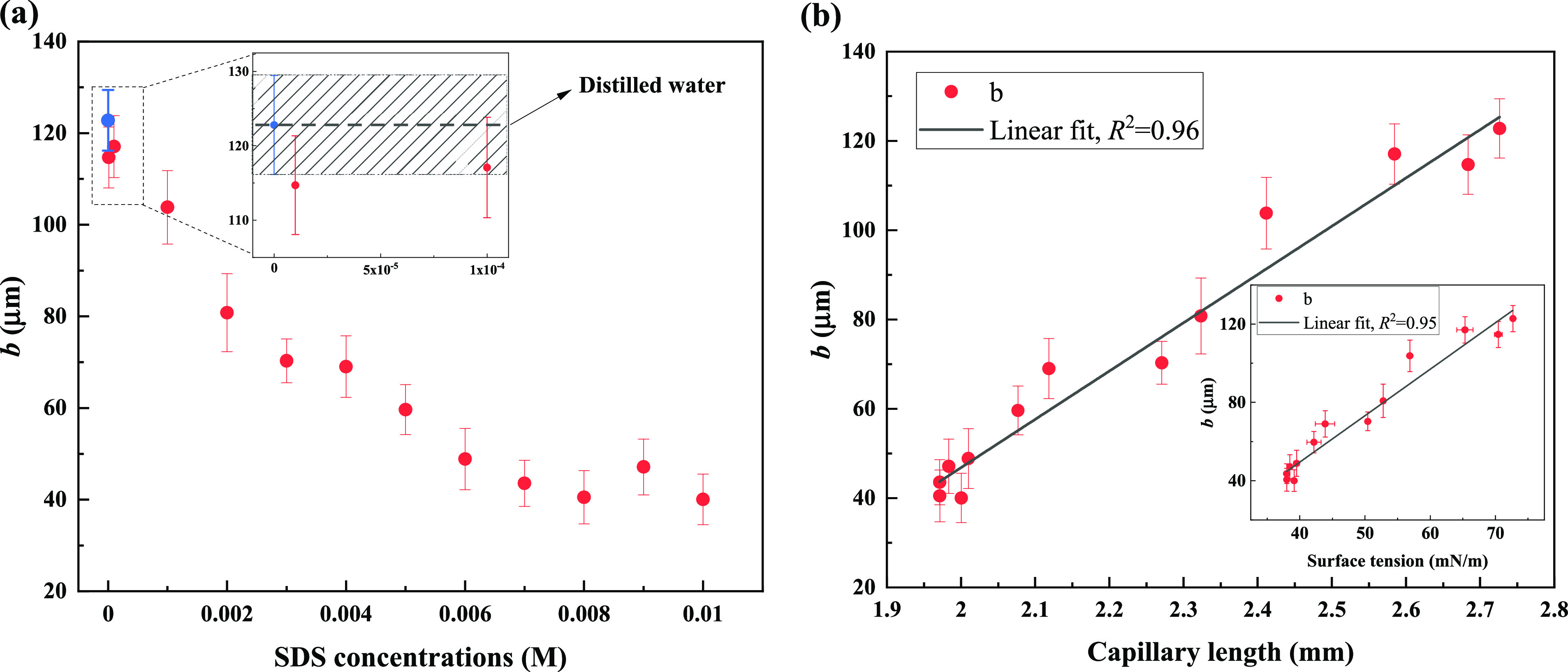
Slip-length measurement on SPNC surfaces with varying SDS solutions. (a) Slip-length results against SDS concentration. The expanded graph shown in panel (a) provides the slip-length values for distilled water (blue filled circle and dashed line, the gray region represents the error of repeats) and “trace” amount of SDS solutions. (b) Slip-length results against the capillary length and surface tension (as an inset) of various SDS solutions. The fitting lines show a clear linear relationship between slip-length and capillary length/surface tension. The plot with capillary length shows a slightly better linear fit than the inset one with surface tension. Error bars represent the variations of repeats (3δ).

SDS solutions were applied on the same SPNC surface for slip-length measurement, and the results are plotted in Figure 5. Consistent with surface tension and contact angle results, the slip length showed a distinctive decreasing trend when SDS concentrations were smaller than the CMC (1–7 mM), becoming approximately constant between 7 and 10 mM, which is above the CMC (Figure 5a). Although a few studies^6,47^ already stated that high contact angle does not necessarily mean a large apparent slip, the slip length (as an indicator of drag reduction) and contact angle (as an indicator of wettability) results have a strong relationship on the present SPNC surfaces. Figure 5a also implies that a Cassie wetting state can be achieved even though contact angles are no longer strictly in the SHO region (i.e., <150°), since the surfaces are still drag-reducing (exhibiting certain slip-lengths) with SDS concentrations larger than 4 mM (above which contact angles are lower than 150°, as shown in Figure 4a). An expanded view in Figure 5a shows that the “trace” amount of surfactant does not reduce significantly the slip length as the difference is within the measurement uncertainty. However, the surface tension of these two liquid samples showed a measurable decrease from water (Figure 3a). This demonstrates for these surfaces that slip-length is not significantly affected by “trace” amounts of surfactants, even though surface tension is measurably lower. It was shown in a recent study that the slip length could be decreased by the surface tension gradient-induced Marangoni force in the circumstances where water has been slightly contaminated by surfactant.^21^ Our current results are not in accord with this. One of the major reasons for this difference could be that an irregularly structured SHO surface was used here, whereas in ref (21), a regular topology of rectangular gratings was used. Therefore, in this case, the surfactant particles are not able to accumulate such that preferential surface tension gradients are formed over the topography and Marangoni stresses are, therefore, not significant.

According to eqs 2 and 3, slip length is predicted to be linearly proportional to the thickness of the air layer (*l*_air_) at a given viscosity ratio (μ_l_/μ_air_). Since liquids with relatively lower surface tension can enter the microstructure of SHO surfaces more easily^20^ and the meniscus/stability of the air pockets is dependent primarily on the surface tension^48^ (or, in nondimensional terms, the capillary number^49^) of the liquid, we make the assumption that the surface tension of a liquid has a positive relationship with *l*_air_. Therefore, the slip length is assumed to be linearly proportional to the so-called capillary length (λ_c_) which is determined by mass density and surface tension as
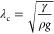
8where γ is the surface tension of the liquid, ρ the mass density of the liquids, and *g* the gravitational acceleration.^50^ λ_c_ is an approximate length scale in a fluid/fluid interface, below which surface tension is able to play a role.^51^ As shown in Figure 5b, a linear fit with *R*^2^ = 0.96 (*R*^2^ is a statistic used to quantify the “goodness-of-fit” and ranges from 0 to 1^52^) was obtained between the measured slip-length and capillary length. For comparison, slip lengths are also plotted as a function of surface tension and are shown in the inset of Figure 5b (linear fit with *R*^2^ = 0.95). Obviously, a larger capillary length (or a higher surface tension) results in a larger slip length. This result is in contradiction with a previous study^20^ in which a *lower* surface tension (i.e., smaller capillary length) was claimed to give a *higher* slip length. However, in their study,^20^ the viscosities of the liquids, which should also play a key role on the slip length, were not kept constant when the effect of surface tension was studied as has been ensured here. It is this lack of isolation of the effect of surface tension from that of viscosity which gives rise to this contradictory result. From a fundamental perspective, the formation of the trapped air (“plastron”) requires a rough surface and a liquid with certain surface tension. A higher surface tension has a tendency to increase the volume of trapped air (i.e., decrease the contact area between solid and liquid^53^) and, therefore, results in a larger apparent slip length. We note that the actual slip length measured is only on the order of a few percent of the magnitude of the capillary length. Additionally, the minimum slip length in this study was found to be 40 μm at a capillary length of 2 mm. This value of slip length is still comparably high, compared with our estimate for its uncertainty (±17 μm, see the uncertainty analysis of slip length in Section S6 in the Supporting Information). Thus, the surface tension of liquids are confirmed to have a direct impact on the slip length of SPNC surface and the surfaces are capable of reducing drag even with surfactant-contaminated water.

### 3.5 Shear Viscosity Dependence of Slip Length

**Figure 6:**
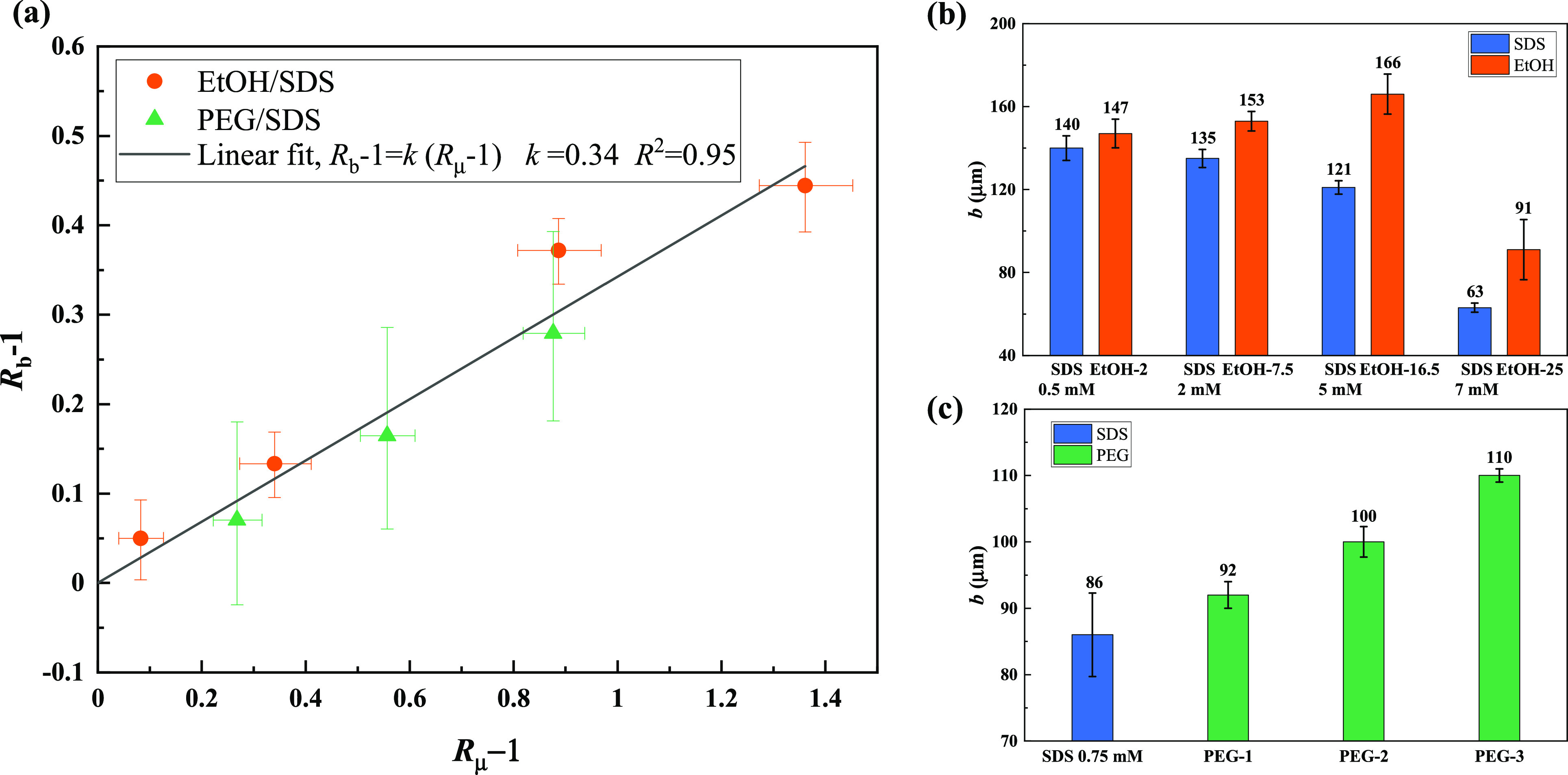
Slip-length measurement on SPNC surfaces with matched fluid samples to keep the surface tension constant. (a) Slip-length ratio minus one (*R*_b_ – 1) versus viscosity ratio minus one (*R*_μ_ – 1) when the surface tensions are matched. A linear fit (*R*^2^ = 0.95) with slope *k* = 0.34 is shown in panel (a), by forcing the intercept to be 0. The slip-length ratio and viscosity ratio for EtOH/SDS are calculated by the results of four concentrations of EtOH solutions divided by the results of four corresponding concentrations of SDS solutions (Figure 3b), whereas for PEG/SDS, the slip length and viscosity ratio are calculated by the results of three concentrations of PEG solutions divided by SDS 0.75 mM (Table 1). The original slip-length data of (b) EtOH/SDS and (c) PEG/SDS are presented as well. The error bar in panel (a) shows the maximum range for viscosity ratio and slip-length ratio (a detailed calculation method can be found in Section S7 in the Supporting Information). Error bars in panels (b) and (c) represent the variations of repeats (3δ).

To solely study the shear viscosity dependence of slip length, surface tension impacts were eliminated by matching with different liquids of equivalent surface tension but differing viscosity. Two sets of slip-length tests were conducted with the surface tension matched EtOH/SDS and PEG/SDS (i.e., the slip-length results from the individual liquids were paired with fluids with identical surface tension), fluid pairs respectively (see Figure 6). The raw slip-length results shown in Figures 6b and 6c were processed into a nondimensional quantity *R*_b_ – 1, which represent the slip-length ratio of two liquids (with the matched surface tension) minus one. Meanwhile, the viscosity ratio minus one (*R*_μ_ – 1) was calculated for the same paired liquids. Shown in Figure 6a is the relationship between *R*_b_ – 1 and *R*_μ_ – 1, and a linear fitting line (*R*_b_ – 1 = *k*(*R*_μ_ – 1), *k* = 0.34) is presented as well. All the data points overlap with the fitting line within the experimental uncertainty. Therefore, the slip length is also revealed to be linearly proportional to the shear viscosity over SPNC surfaces when surface tension differences are properly taken into account. Figure 6a also implies that the slip lengths increase with the increasing viscosity of the liquid because a higher liquid viscosity results in a higher μ_l_/μ_air_, as described in eqs 2 and 3. Furthermore, increasing the viscosity has less impact on slip length than in the limited results of Choi and Kim^18^ and Ahmmed et al.,^19^ as doubling the viscosity only results in a 34% increase in slip length (the slope of the fit shown in Figure 6a is 0.34). Both studies^18,19^ reported that an ∼2.5-fold increase in viscosity will lead to the slip length increasing by a factor of ∼2.5 with the same measurement technique but different SHO materials and microgeometries. Their results are consistent with the simplistic theoretical prediction (eqs 2 and 3), as the slip-length ratio should be equal to the viscosity ratio.^33^ (However, more involved theoretical predictions that allow spatially dependent partial slip,^34^ indicate a potential saturation of the local slip lengths or, including meniscus curvature, exhibit more complex variations with viscosity contrast:^54^ a lack of detailed surface topology information precludes a more detailed comparison to these interesting theoretical studies here). However, in their studies, only slip lengths of 10–30 μm with water were obtained, which is close to the sensitivity of the rheometer system used^55^ (we estimate our uncertainty to be ±17 μm). We note that the surface tension of the liquids used in Choi and Kim^18^ and Ahmmed et al.^19^ could have also influenced the slip length, but this effect was not considered in both papers (the increased viscosity fluids in these studies have lower surface tensions than water and so the “true” influence of viscosity alone would be greater than linear). In the current study, the minimum slip length of the PEG experiments was measured to be 86 μm (Figure 6c) and the surface tension of liquids was controlled to be constant for a fair comparison. This is significantly bigger than our uncertainty in slip-length estimation (which is ∼20 μm). Meanwhile, a very consistent trend was obtained from the results of both EtOH/SDS and PEG/SDS. Therefore, it is concluded that the dependence of slip length on shear viscosity does exist with this irregularly structured SPNC surface, but it is lower than the prediction of the idealized view encapsulated in eqs 2 and 3.

## 4 Conclusions

Since most of the previous studies in the literature have focused on the effect of material and microgeometrical impacts on the slip length of SHO surfaces, the present research provides an insight into the influence of liquid properties. The surface tension and shear viscosity dependence of slip length were systematically investigated by using various liquids (SDS, EtOH, and PEG) and a SHO surface (SPNCs), which provides significant apparent slip.

Because of the decreasing surface tension via the addition of a surfactant (SDS), the SHO property of the SPNCs is significantly reduced with a decreasing contact angle. However, SPNC surfaces are able to remain in the hydrophobic region and exhibit drag reduction characteristics with an SDS concentration larger than the CMC. With the constant viscosity of SDS solutions, slip lengths are shown to be linearly proportional to the capillary length. Based on this, we hypothesize that the surface tension mainly affects the air layer thickness via the capillary length, and, therefore, decreasing the surface tension causes a decrease in air layer thickness and slip length, as the theoretical prediction, *b* ∝ *l*_air_. The relationship between slip length and shear viscosity when surface tension is held fixed is shown to be linearly proportional as well. Results are consistent between different liquids/fluid pairs. Moreover, we should note that, in the current study, the slip-length increase ratio does not follow the viscosity increase ratio which indicates the idealized view of apparent slip (e.g., eq 2 and 3) may not fully hold for irregularly structured SHO surfaces.

## Supporting Information Available

Shear-rate settings and Reynolds number, SEM images of different SPNC samples, slip-length characterization of SPNC surfaces, time endurance experiments for SPNC surfaces, examples for the viscosity results, uncertainty analysis of slip length, as well as error bar determination method in Figure 6a (PDF)
