# Supplementary material for: Surface Tension and Viscosity Dependence of Slip Length over Irregularly Structured Superhydrophobic Surfaces
Source: Langmuir. 2022 Sep 20;38(39):11873–81. doi: 10.1021/acs.langmuir.2c01323 (PMC9536016; doi:10.1021/acs.langmuir.2c01323)
Supplement: Supplementary file 1 — la2c01323_si_001.pdf [file la2c01323_si_001.pdf]

Supporting Information

Surface tension and viscosity dependence of slip-length over irregularly-structured superhydrophobic surfaces

Linsheng Zhang[a], *Yasmin A. Mehanna*[b,c], *Colin R. Crick*[c], *and Robert J. Poole*[a,*]

a School of Engineering, University of Liverpool, Liverpool, L69 3GH, United Kingdom

b Materials Innovation Factory, Department of Chemistry, University of Liverpool, Liverpool, L69 7ZD, United Kingdom

c School of Engineering and Materials Science, Queen Mary University of London, London, E1 4NS, United Kingdom

* Corresponding author Email: robpoole@liverpool.ac.uk

S1. Shear-rate settings and Reynolds number

Table S1. Shear-rate used for slip-length measurement along with the corresponding shear stress and Reynolds numbers

Samples	Shear-rate (1/s)	Shear stress[a] (Pa)	Reynolds number[b]
Distilled water	50-100	0.05-0.1	40-56
SDS solutions	50-100	0.05-0.1	40-56
PEG-1	60	0.07	39
PEG-2	50	0.08	32
PEG-3	40	0.07	26
EtOH-2	75	0.08	47
EtOH-7.5	60	0.08	38
EtOH-16.5	40	0.07	26
EtOH-25	30	0.07	20

[a]Shear stress obtained from the no-slip reference setup. The shear stress measured on a SHO surface will be lower.

[b]Reynolds number (Re) was calculated via $Re = R\sqrt{\rho\omega/\mu}$, where R is the diameter of the cone, ρ is the mass density of the liquid, ω is the angular velocity, and μ is the viscosity of the liquid[1].

S2. SEM images of different SPNC samples

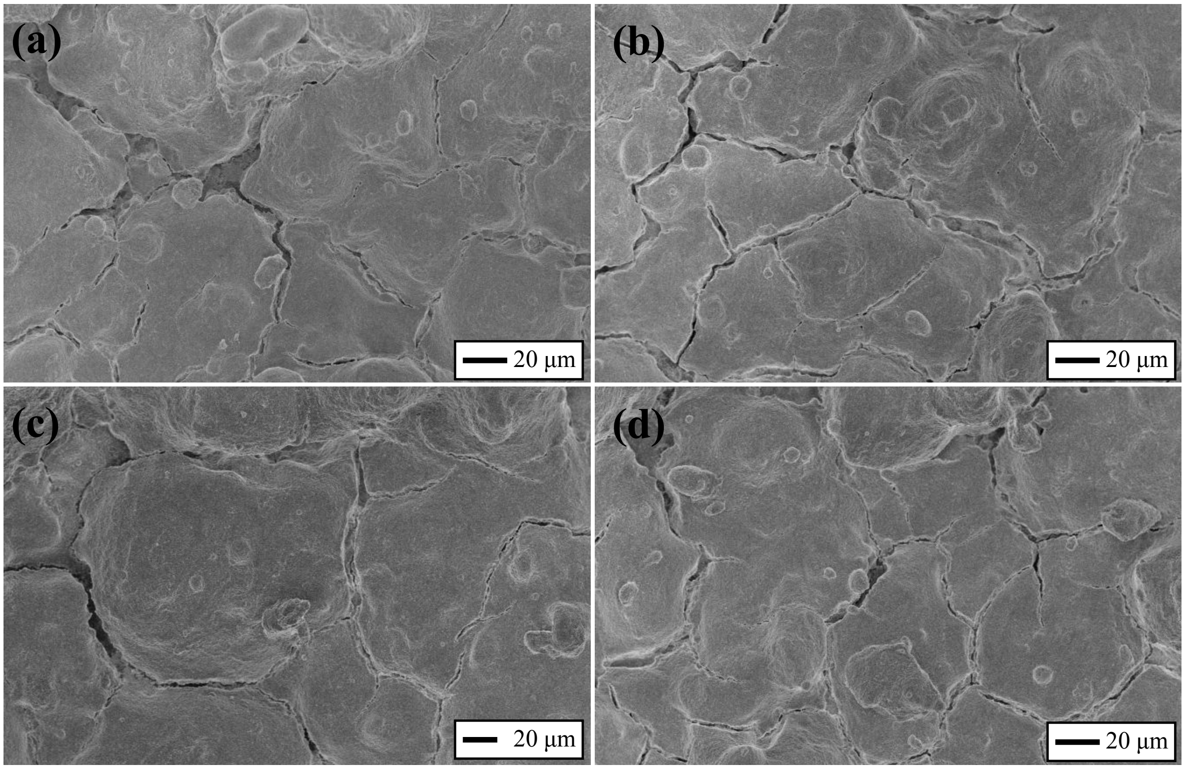

Figure S1. SEM images of different SPNC samples at different locations. (a) Sample 1, Location 1. (b) Sample 1, Location 2. (c) Sample 2, Location 1. (d) Sample 2, Location 2. Scale bars are shown for each image.

S3. Slip-length characterisation

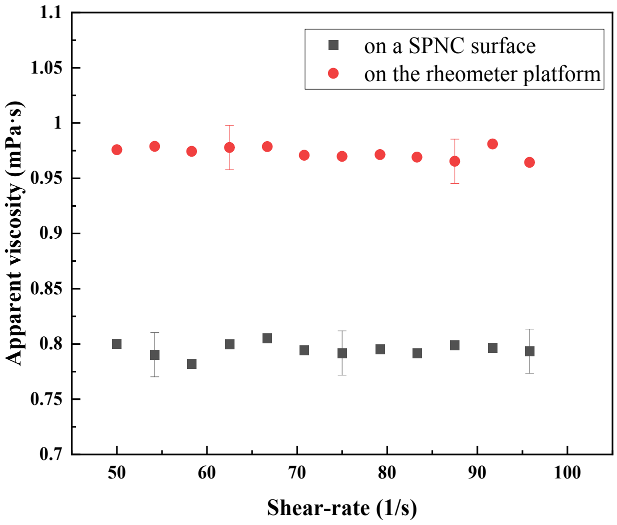

Figure S2. Slip-length characterisation for a SPNC surface with distilled-water. The apparent viscosity of distilled-water at 20°C was measured to be 0.97 mPa·s on the rheometer platform and 0.80 mPa·s on a SPNC surface. Therefore, the slip-length for the current surface is 154 μm, equivalent to 17.5% drag-reduction in the range *Re*=40-56. Error bars show the standard deviation of repeat measurements.

S4. Time endurance experiments for SPNC surfaces

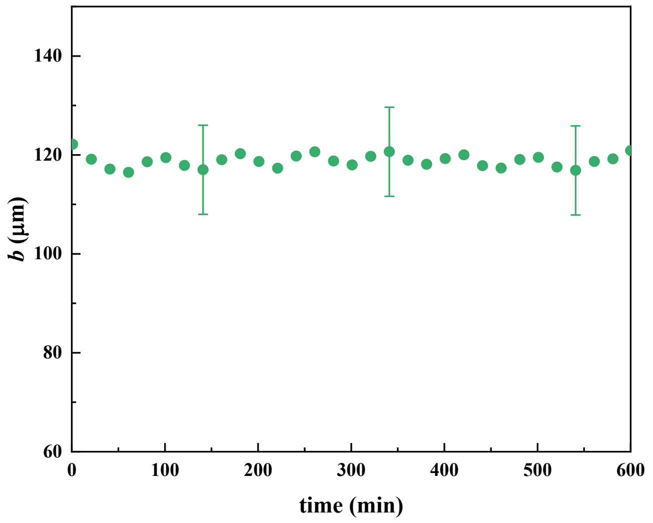

Figure S3. Long-term slip length measurement on a SPNC surface. The test was conducted over a period of 10 hours with distilled-water at a shear-rate of 75 1/s. The variation of slip-length during this time were observed to be within experimental uncertainty.

S5. Examples for the viscosity results

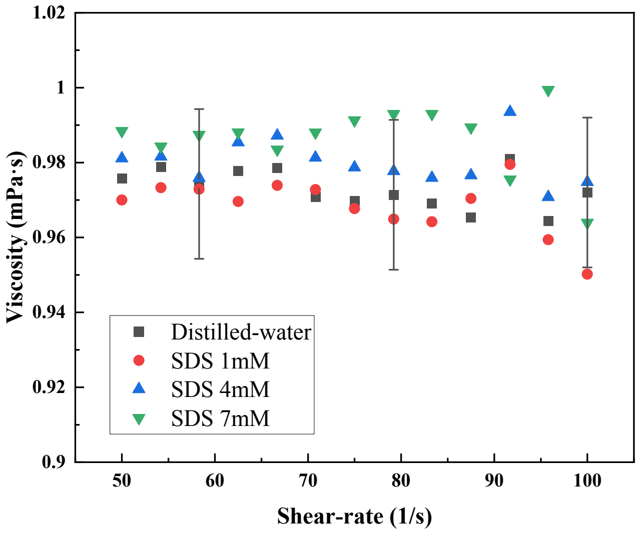

Figure S4. Viscosity results of distilled-water and various SDS solutions. Error bars shows the variations of repeats. The viscosities of three SDS solutions (1, 4 and 7 mM) have no measurable difference with the viscosity of distilled-water. All the viscosity results lie within experimental uncertainty (0.97±0.02 mPa·s).

S6. Uncertainty analysis of slip-length

The potential errors involved in the determination of the slip-length result from three main factors: instrument uncertainty, inertial effects, and edge effects. Due to the low shear rate (<100 1/s) and Reynolds number (Re<56), significant inertial effects are avoided as no obvious secondary flow occurred during the measurements[2] (i.e., no increased viscosity is observed at the higher shear rates). Any edge effect is reduced significantly by matching the diameter of the SHO surface and the rheometer cone. In addition, the free surface can be observed visually to ensure the curvature is relatively constant since the filled water is visible as the cone is transparent.

The previous discussion confirms the inertial and edge effect are limited by the experimental operations so that the major uncertainty comes from the rheometer uncertainty. The rheometer (Anton Paar MCR 302) employed has a quoted resolution of 0.1 nN·m which provides a negligible error (<0.005%) at the torque range 2-10 μN·m for all the slip length experiments. As stated by the manufacturer, the gap sizing error of the current rheometer is less than 0.6 μm (confirmed with the service engineer from Anton Paar Ltd.). Therefore, the reference torque without slip are considered to overlook the gap error as zero-gap was configured as 118 μm. In addition to the systematic gap error, the fabricated SHO surface are estimated to have a thickness of 30±10 μm. Combined with

the error of the circular substrate thickness (±10 μm), the overall uncertainty of gap would be ±20 μm, resulting in a torque error $\Delta T/T = 1.7\%$ with an SHO surface. Meanwhile, the temperature variation of liquid samples (20±0.5℃) contributes a maximum torque uncertainty of 1.5%. Thus, the uncertainty of torque is less than 2.3%, equivalent to a slip length of ±17 μm.

S7. Error bar determination method (**Figure 6a)**

To present the complete range of viscosity ratio (R_{μ}) and slip-length ratio (R_{b}), the raw data are processed by the following method to determine the error bars in **Figure 6a**. Assuming the average viscosities and uncertainties of a pair of liquids (e.g., EtOH-2/SDS 0.5 mM) were $\mu_1 \pm x_1$ and $\mu_2 \pm x_2$ ($\mu_1 > \mu_2$) mPa·s respectively (i.e., $\mu_1$was viscosity of EtOH-2 and μ_2 was viscosity of SDS 0.5mM). Meanwhile, the average slip-lengths and uncertainties using these two liquids were assumed to be $b_1 \pm y_1$ and $b_2 \pm y_2$ ($b_1 > b_2$) μm respectively (i.e., $b_1$was slip-length result received from EtOH-2 and b_2 was viscosity slip-length result received from SDS 0.5mM). Then the upper limit of viscosity ratio (R_{μ}^{ul}) was calculated via,

$$R_{\mu}^{ul} = \frac{\mu_1 + x_1}{\mu_2 - x_2} \#(S1)$$

The lower limit of viscosity ratio (R_{μ}^{ll}) was calculated via,

$$R_{\mu}^{ll} = \frac{\mu_1 - x_1}{\mu_2 + x_2} \#(S2)$$

Similarly, the upper and lower limit of slip-length ratio (R_{b}^{ul} and R_{b}^{ll}) were determined by **Equation S3** and **S4** respectively,

$$R_{b}^{ul} = \frac{b_1 + y_1}{b_2 - y_2} \#(S3)$$

$$R_{b}^{ll} = \frac{b_1 - y_1}{b_2 + y_2} \#(S4)$$

Thus, the error bar of $R_{\mu} - 1$ and $R_{b} - 1$ were determined via the corresponding upper and lower limits minus one.

References

1. Imayama, S.; Alfredsson, P. H.; Lingwood, R. J., An experimental study of edge effects on rotating-disk transition. *Journal of Fluid Mechanics* **2013,** *716*, 638-657.

2. Imayama, S.; Alfredsson, P. H.; Lingwood, R. J., On the laminar–turbulent transition of the rotating-disk flow: the role of absolute instability. *Journal of Fluid Mechanics* **2014,** *745*, 132-163.
